# Real-time analysis of hospital length of stay in a mixed SARS-CoV-2 Omicron and Delta epidemic in New South Wales, Australia

**DOI:** 10.1186/s12879-022-07971-6

**Published:** 2023-01-17

**Authors:** Ruarai J. Tobin, James G. Wood, Duleepa Jayasundara, Grant Sara, Camelia R. Walker, Genevieve E. Martin, James M. McCaw, Freya M. Shearer, David J. Price

**Affiliations:** 1 Melbourne School of Population and Global Health, The University of Melbourne, Melbourne, Australia; 2 School of Population Health, University of New South Wales, Sydney, Australia; 3 System Information and Analytics Branch, New South Wales Ministry of Health, Sydney, Australia; 4 Northern Clinical School, Faculty of Medicine and Health, University of Sydney, Sydney, Australia; 5 School of Mathematics and Statistics, The University of Melbourne, Melbourne, Australia; 6 Department of Infectious Diseases, Melbourne Medical School, The University of Melbourne, Melbourne, Australia; 7 Department of Infectious Diseases, Central Clinical School, Monash University, Melbourne, Australia; 8 Doherty Institute for Infection and Immunity, The Royal Melbourne Hospital and The University of Melbourne, Melbourne, Australia

**Keywords:** COVID-19, Survival analysis, Multi-state model, Length of stay

## Abstract

**Background:**

The distribution of the duration that clinical cases of COVID-19 occupy hospital beds (the ‘length of stay’) is a key factor in determining how incident caseloads translate into health system burden. Robust estimation of length of stay in real-time requires the use of survival methods that can account for right-censoring induced by yet unobserved events in patient progression (e.g. discharge, death). In this study, we estimate in real-time the length of stay distributions of hospitalised COVID-19 cases in New South Wales, Australia, comparing estimates between a period where Delta was the dominant variant and a subsequent period where Omicron was dominant.

**Methods:**

Using data on the hospital stays of 19,574 individuals who tested positive to COVID-19 prior to admission, we performed a competing-risk survival analysis of COVID-19 clinical progression.

**Results:**

During the mixed Omicron-Delta epidemic, we found that the mean length of stay for individuals who were discharged directly from ward without an ICU stay was, for age groups 0–39, 40–69 and 70 +, respectively, 2.16 (95% CI: 2.12–2.21), 3.93 (95% CI: 3.78–4.07) and 7.61 days (95% CI: 7.31–8.01), compared to 3.60 (95% CI: 3.48–3.81), 5.78 (95% CI: 5.59–5.99) and 12.31 days (95% CI: 11.75–12.95) across the preceding Delta epidemic (1 July 2021–15 December 2021). We also considered data on the stays of individuals within the Hunter New England Local Health District, where it was reported that Omicron was the only circulating variant, and found mean ward-to-discharge length of stays of 2.05 (95% CI: 1.80–2.30), 2.92 (95% CI: 2.50–3.67) and 6.02 days (95% CI: 4.91–7.01) for the same age groups.

**Conclusions:**

Hospital length of stay was substantially reduced across all clinical pathways during a mixed Omicron-Delta epidemic compared to a prior Delta epidemic, contributing to a lessened health system burden despite a greatly increased infection burden. Our results demonstrate the utility of survival analysis in producing real-time estimates of hospital length of stay for assisting in situational assessment and planning of the COVID-19 response.

**Supplementary Information:**

The online version contains supplementary material available at 10.1186/s12879-022-07971-6.

## Background

The state of New South Wales (NSW), Australia, employed a broadly successful suppression strategy against COVID-19 for the early period of the pandemic, with relatively low case incidence prior to an outbreak of the Delta variant of SARS-CoV-2 (PANGO lineage B.1.617.2) in June 2021. This Delta epidemic grew substantially despite stepped escalation of public health and social measures, supported by intensive test-trace-isolate-quarantine measures. Cases totalled to 53,851 notifications across a two month period of August and September 2021, with a peak in hospital occupancy of 1,268 beds on 21 September. Subsequently, daily cases continued to decline, reaching a plateau in the low hundreds by late-October 2021 as the effective reproduction number stabilised below 1—in part due to substantial vaccination uptake [1].

**Fig. 1:**
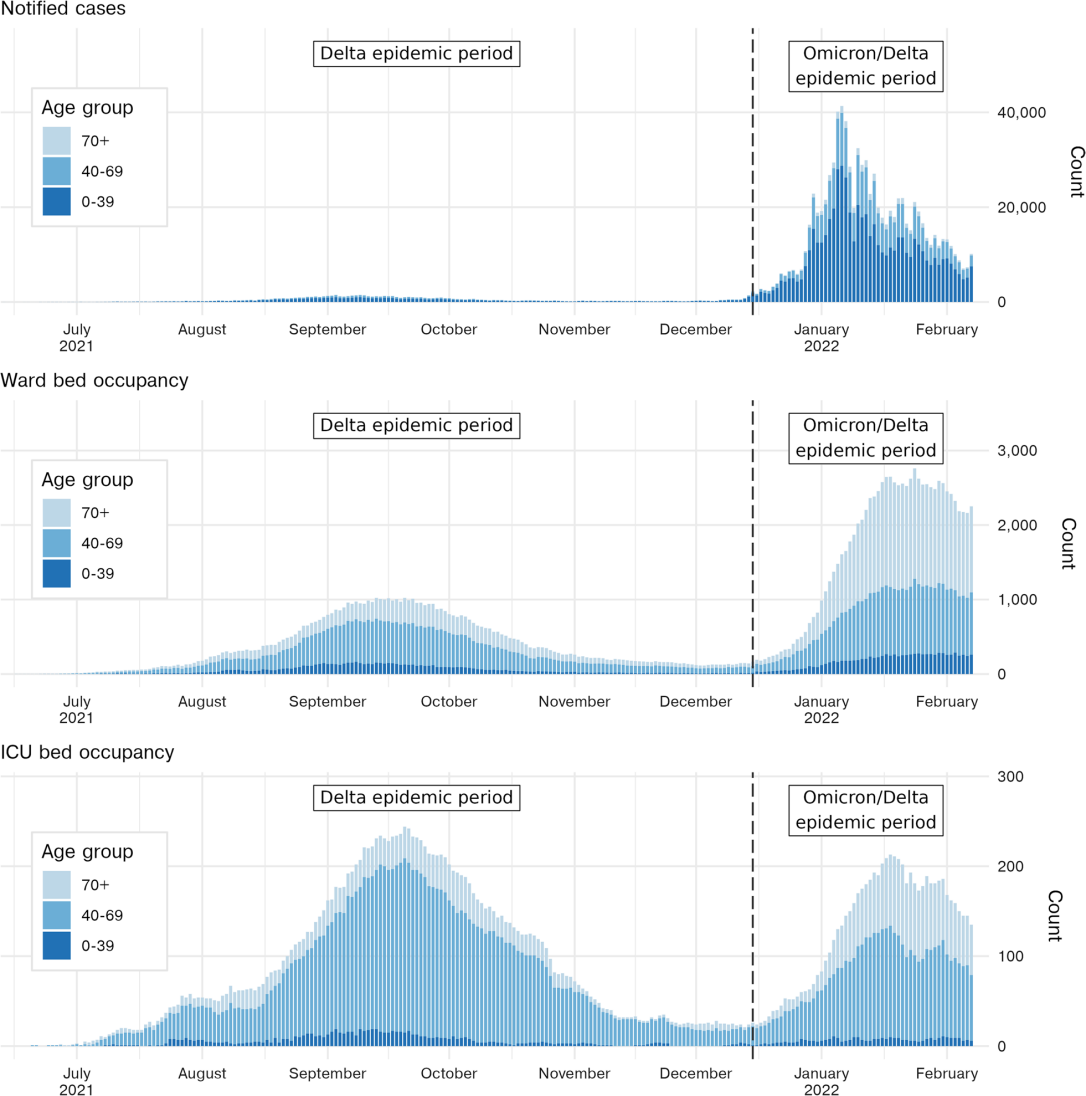
Number of COVID-19 cases by notification date and count of ward and ICU beds occupied by COVID-19 patients across the New South Wales hospital system. Case notification data is via Data.NSW and the NSW Ministry of Health [34, 35], hospital occupancy data via NSW Ministry of Health Patient Flow System [8]. The number of notified cases may differ from counts reported at that date where later deduplication or reclassification of cases has been performed. Plotted data ranges from 1 July 2021 to 15 February 2022, with the dashed vertical line indicating the end date of the Delta epidemic period/start date of the mixed Omicron-Delta epidemic period for the purposes of this analysis

The SARS-CoV-2 Omicron variant (lineage BA.1) was first identified in the New South Wales population on 28 November 2021 [2–4], with significant transmission including super-spreading events occurring in the following four weeks [5, 6]. Notified COVID-19 cases continued to increase sharply, from 288 on 1 December 2021 up to 2250 on 15 December. Over the period of 10–24 January, Omicron represented 94% of sequenced cases in Australia [3, 7]. A total of 826,942 case notifications were made across a two month period from December 2021 through January 2022, which translated into a peak hospital occupancy of 2943 reported on 25 January. Figure 1 shows case notifications and the reported ward and ICU occupancy through the study periods by age group.

Robust estimates of the hospital length of stay are essential to understanding the impact of disease on hospital burden, and thus to support decision making. Conducting such analyses in real-time, particularly during early stages of disease emergence (including novel variants), is paramount for providing up-to-date and timely advice for planning. Given the nature of a patients progression through the hospital system, such real-time analyses will likely consist of some patients who are still in the hospital, where their next transition (e.g., discharge, transfer to ICU, death), and the time at which it will occur, are as yet unknown. Generating robust estimates of the length of stay and transition probabilities in the presence of this right-censoring requires appropriate statistical methods, designed to account for such occurrences.

Using data available as of 7 February 2022, we examined the length of stay distributions of hospitalised COVID-19 cases in NSW in two periods: the Delta only epidemic (1 July 2021–14 December 2021) and the mixed Omicron and Delta epidemic (15 December 2021–7 February 2022) to estimate the reduction in hospital length of stay during these periods. For the latter period, we further examined length of stay for a subset of cases hospitalised in the Hunter New England Local Health District of NSW where Omicron was reportedly the dominant, if not only, circulating variant. These time windows were chosen to characterise the outbreaks of Delta and Omicron variants in the absence of sufficient patient-level variant data. We performed an age-specific competing-risk survival analysis using a compartmental model of COVID-19 clinical progression, and report parameters for estimated length of stay distributions and corresponding summary statistics. This framework, which could fully utilise the censored data available at the time, allowed for our results to inform clinical forecasting efforts and policy decision making in real-time.

## Methods

### Data

Episode level hospital stay data of COVID-19 patients was provided by the NSW Ministry of Health extracted from the Patient Flow System [8]. Each datum consists of a singular episode within a ward with associated dates and times of admission and discharge. In order to provide reliable estimates of the length of stay of patients, we performed several filtering steps to the data prior to fitting the multi-state model. Where an individual had been admitted to the intensive care unit (ICU), the initial and final known date and time were available in the data, alongside a total duration within ICU (which excluded any periods of ward stay between these initial and final known dates, and may be indicative of readmission to ICU). Where the recorded duration in ICU was less than that indicated by the admission and discharge dates and times (which differed in only approximately 6% of admissions), we assumed that the additional time outside the ICU contributed to the post-ICU ward stay. This ensured—within the limitations of the compartmental model—that any ward stay that occurred during the period from a patient’s initial to final ICU date would not contribute to our estimates of ICU length of stay.

In addition, the data contained the date of symptom onset and age for each patient. As we did not have access to information on each individual’s vaccination status, previous SARS-CoV-2 infections, comorbidities, or treatments received, we were not able to investigate the potential effects of these factors in our analysis.

**Fig. 2:**
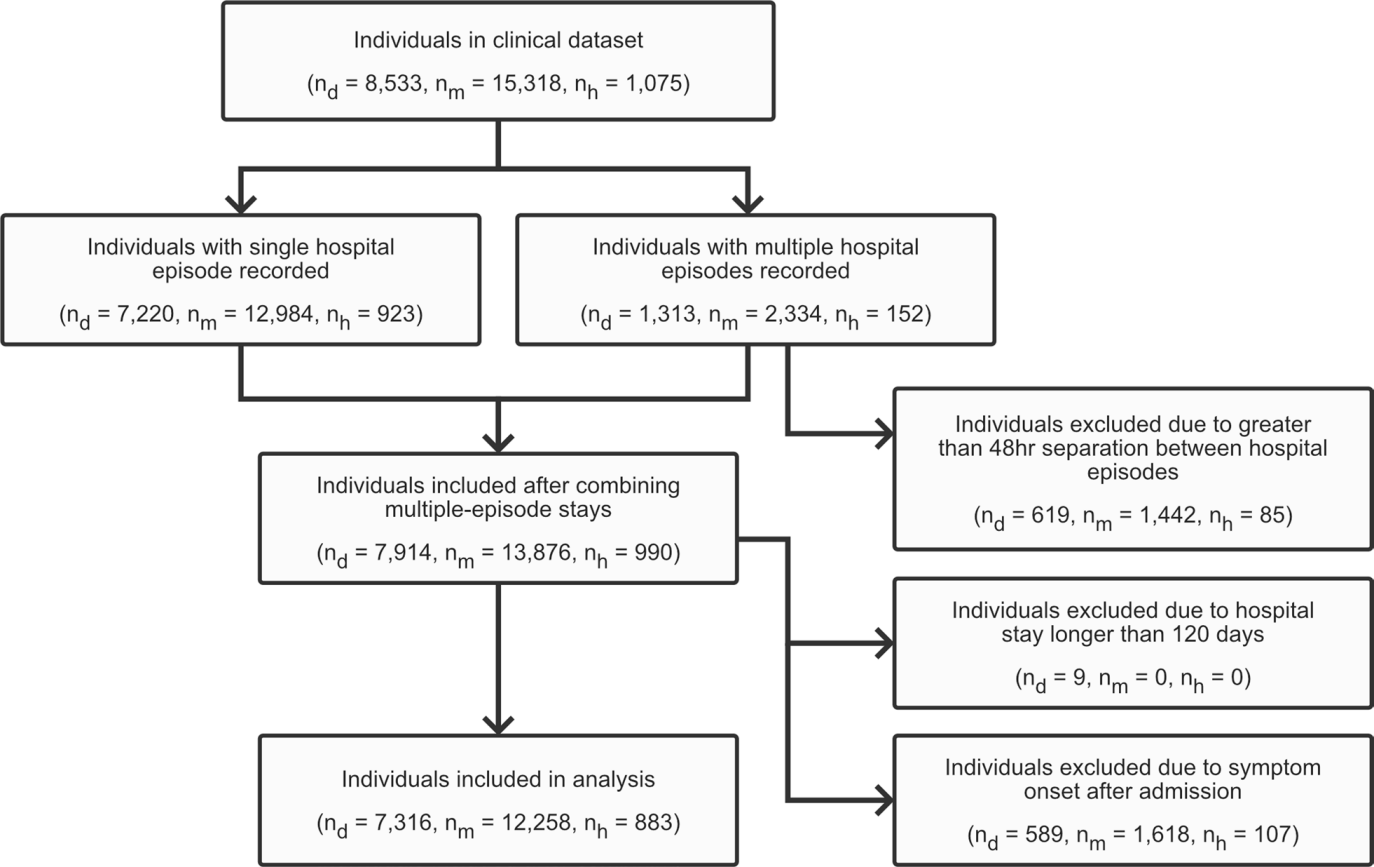
Data inclusion flowchart, where n$_d$, n$_m$ and n$_h$ is the number of individuals in the Delta epidemic period, mixed Omicron-Delta epidemic period and Omicron Hunter New England epidemic period analysis respectively. Note that the individuals included in the Omicron Hunter New England analysis are also included in the mixed Omicron-Delta analysis

As an individual could have numerous episodes across multiple wards (e.g. representing transfer between different wards), episodes were concatenated to produce a single stay, where an individual would be assumed to be in hospital from the time of their earliest episode’s admission until the time of their latest episode’s discharge. At this stage, individuals were excluded from analysis where any two of their consecutive episodes were separated by at least 48 hours outside of the hospital system (n = 619, 1442 and 85 for Delta, mixed Omicron-Delta and HNE Omicron epidemic periods respectively; Fig. 2). This filtering ensured that all cases included were valid under the single-stay assumption of the compartmental model.

Cases were removed from analysis where symptom onset was recorded to have occurred later than their earliest admission to hospital, given that that such infections were likely to have occurred within the hospital (n = 589, 1618 and 107 for Delta, mixed Omicron-Delta and HNE Omicron epidemic periods respectively; Fig. 2). Although such within hospital infections could be equally likely to progress to a clinically severe state, the lack of differentiation in COVID-19 severity available to our analysis required the removal of these cases. A sensitivity analysis was performed to confirm that estimated lengths of stay were robust to this filtering assumption (Additional file 1: Fig. S4).

Individuals with a total hospital stay duration of greater than 120 days were removed from analysis (n = 9, 0 and 0 for Delta, mixed Omicron-Delta and HNE Omicron epidemic periods respectively; Fig. 2) as these were expected to be more likely to be incidental infections, with this assumption validated by examination of the ward and sub-ward they were recorded as occupying. Furthermore, it was expected that such substantial stay durations (even in the case of them being true sequelae of infection) would have a disproportionate effect in the fitting of the parametric distributions to length of stay.

### Multi-state survival model

**Fig. 3:**
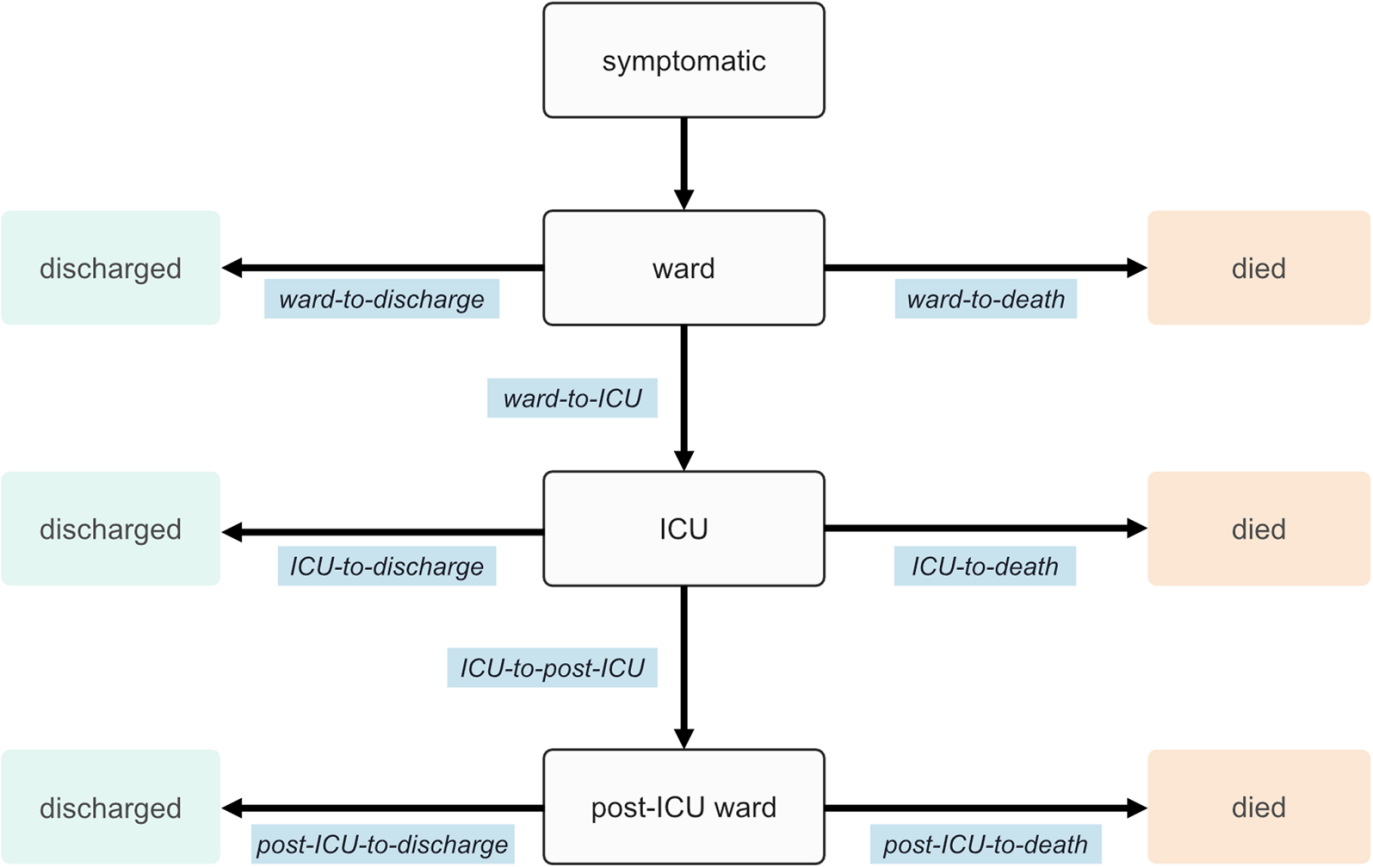
Compartmental model of COVID-19 clinical progression, with length of stay time distributions to be estimated across model transitions displayed in blue

We performed an age-specific competing-risk survival analysis using a compartmental model of COVID-19 clinical progression (Fig. 3). This model framework has been used for similarly characterising hospital demand for other patients infected with other SARS-CoV-2 variants [9, 10] and has been shown to produce reliable estimates of length of stay during an epidemic [11, 12].

At the time of analysis (data up to 7 February 2022), 11% of individuals in the mixed Omicron and Delta epidemic period data had not yet been observed to have an outcome (e.g. at the ward stage, individuals who have been admitted and not yet been discharged, transferred to ICU or died). If this censoring was not appropriately considered in the analysis, estimates would be biased down by an over-representation of individual’s with a shorter length of stay [11]. We considered each branching step in the clinical pathway model as a set of competing risks in a multi-state survival model to account for this censoring. For example, consider admission to the ward upon presenting to the hospital: individuals can transition from ward-to-discharge, ward-to-death or ward-to-ICU. Individuals in the data who were yet to be observed following any of these were then considered to be censored. We utilised a mixture distribution framework, which estimates a multinomial distribution describing the probability that each given transition will occur, then conditional on each probability of transition, subsequently estimates the corresponding parametric distribution describing the length of stay [13]. This competing-risk approach produces time-to-event and transition probability results that are more straightforward to interpret and to utilise in further modelling work in comparison to the more typical cause-specific hazards approach to analysing multi-state survival data [12, 14].

Model estimates were produced using the flexsurv package [14] in R [15], with mixture distribution fits produced across each compartment (ward, ICU, post-ICU-ward). Estimates were stratified by age groups as appropriate to the sample size available for fitting (i.e., broader age grouping used where sample size was too low to produce reliable estimates). The distributions of lengths of stay were modelled as gamma distributions across all transitions, with shape and rate parameters varying by age group in the ward compartment, but with only the shape parameter varying by age group in the ICU and post-ICU-ward compartments (due to limited sample size). To improve model fit for the ward-to-death and post-ICU-to-death pathways, fixed probabilities of transition were specified according to the non-parametric Aalen-Johansen estimates. Cumulative survival probability plots were produced across each pathway, comparing the parametric gamma distribution fits with non-parametric Aalen-Johansen estimates to visually assess goodness-of-fit (Additional file 1: Figs. S2, S3).

To evaluate the performance of the real-time estimation process, we performed two additional analyses: (1) ‘retrospective’ length of stay estimates were produced using a later data extract (29 August 2022 compared to 7 February 2022), by which time all individuals admitted during the mixed Omicron-Delta and Omicron HNE epidemic periods had complete observations and (2) ‘naive’ length of stay estimates were produced using data as at 7 February, removing any individuals with censored observations. As such, the first analysis allows us to compare the length of stay estimates for these now fully-observed patients against the real-time estimates which had as yet unobserved events and event times in the data. The second analysis allows us to demonstrate the benefit of incorporating censored observations in an analysis of the hospital length of stay. For each of these analyses, distribution fits were produced with the fitdistrplus R package [16].

## Results

**Fig. 4:**
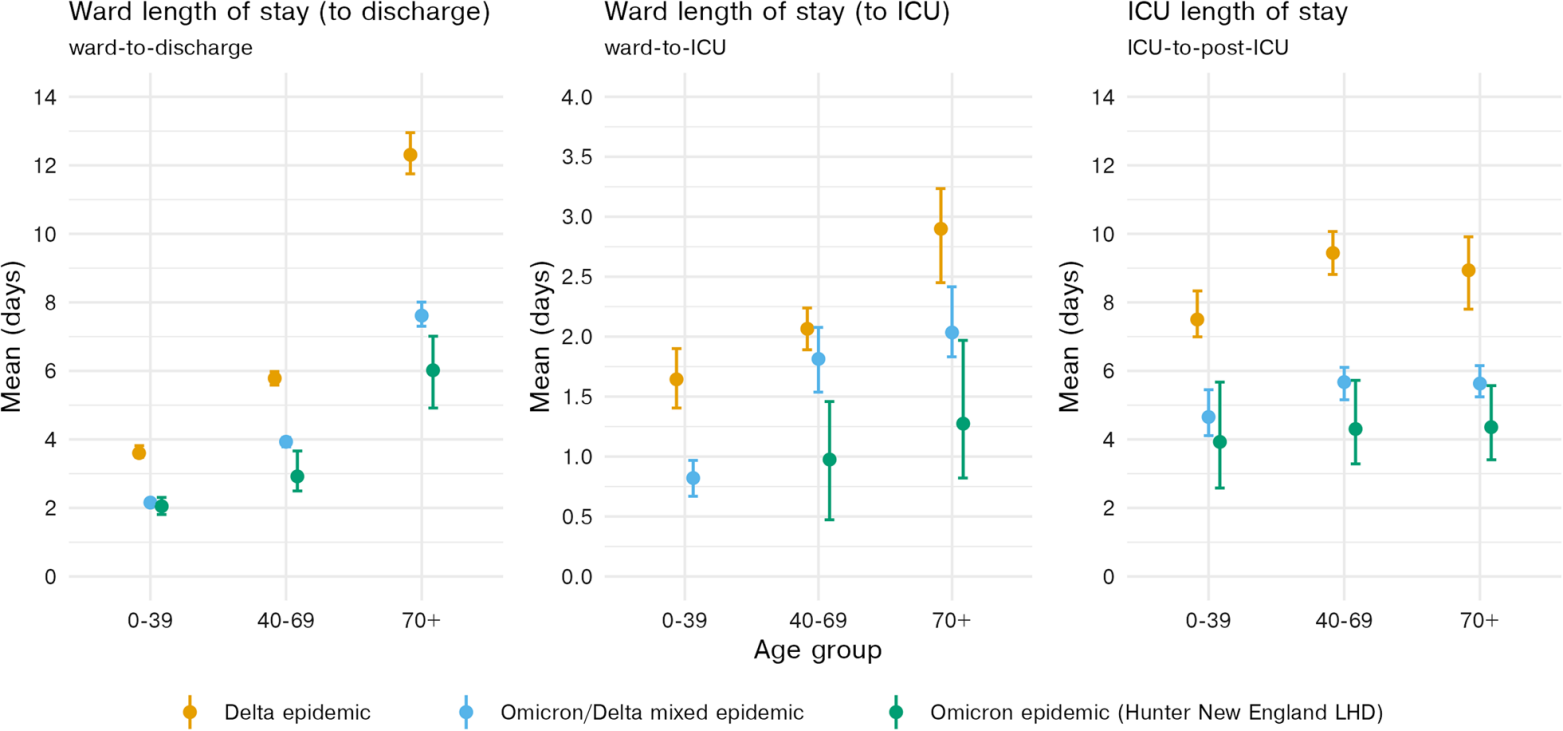
Modelled mean length of stays for the ward-to-discharge, ward-to-ICU and ICU-to-post-ICU pathways. Means and 95% confidence intervals are shown. Note that the y-axis differs between panels. Delta estimates are produced over individuals admitted to hospital between 1 July 2021 and 14 December 2021. Omicron and mixed Omicron-Delta estimates are produced over individuals admitted to hospital between 15 December 2021 and 7 February 2022. Length of stay estimates for the ICU pathway do not include patients who are recorded as having been discharged directly from the ICU (i.e. not including the direct ICU-to-discharge pathway). Estimates from the Hunter New England Omicron dataset are not displayed where sample size was insufficient to produce a model fit

We estimated that the mean length of stay for patients hospitalised during the mixed Omicron and Delta epidemic period (15 December 2021–7 February 2022) was reduced by a factor of approximately half across most clinical pathways compared to the Delta-only period (1 July 2021–14 December 2021) (Fig. 4, Table 1). Figure 4 shows that the estimated mean lengths of stay across all age groups and transitions were shorter again when restricting the analysis to cases hospitalised within the Hunter New England local health district (LHD), where it was reported that Omicron was almost certainly the only circulating variant. As has been observed previously, the increase in mean length of stay by age is present for each period for stays in the ward (e.g., [10]).

Additional file 1: Table S1 contains the estimated mean length of stay and upper 90th-percentiles (with 95% confidence intervals) for each transition by age group and during each of the Delta, mixed Omicron-Delta and Omicron epidemics. Additional file 1: Table S1 reports the corresponding scale and shape parameters, the distribution medians, their 95% confidence intervals, the correlation between the parameter estimates, and probabilities of transitions between model compartments. Additional file 1: Fig. S1 further demonstrates the correlation between bootstrapped shape and scale parameter estimates.

The probabilities reported in Additional file 1: Table S1 are estimated as described above, and are not adjusted for comorbidities, vaccination status, prior infection with SARS-CoV-2, sex, or other variables associated with severity of disease (e.g., [17, 18]). These are estimates of the observed transition probabilities during the mixed Omicron-Delta epidemic in the largely previously uninfected study population over a specific time period—they do not represent a formal severity analysis. We caution against generalising these values to other settings, particularly with different demographics, vaccination coverage, models of clinical care, or prevalence of comorbidities, or assuming that these probabilities remain constant in other time-periods in the study population.

**Fig. 5:**
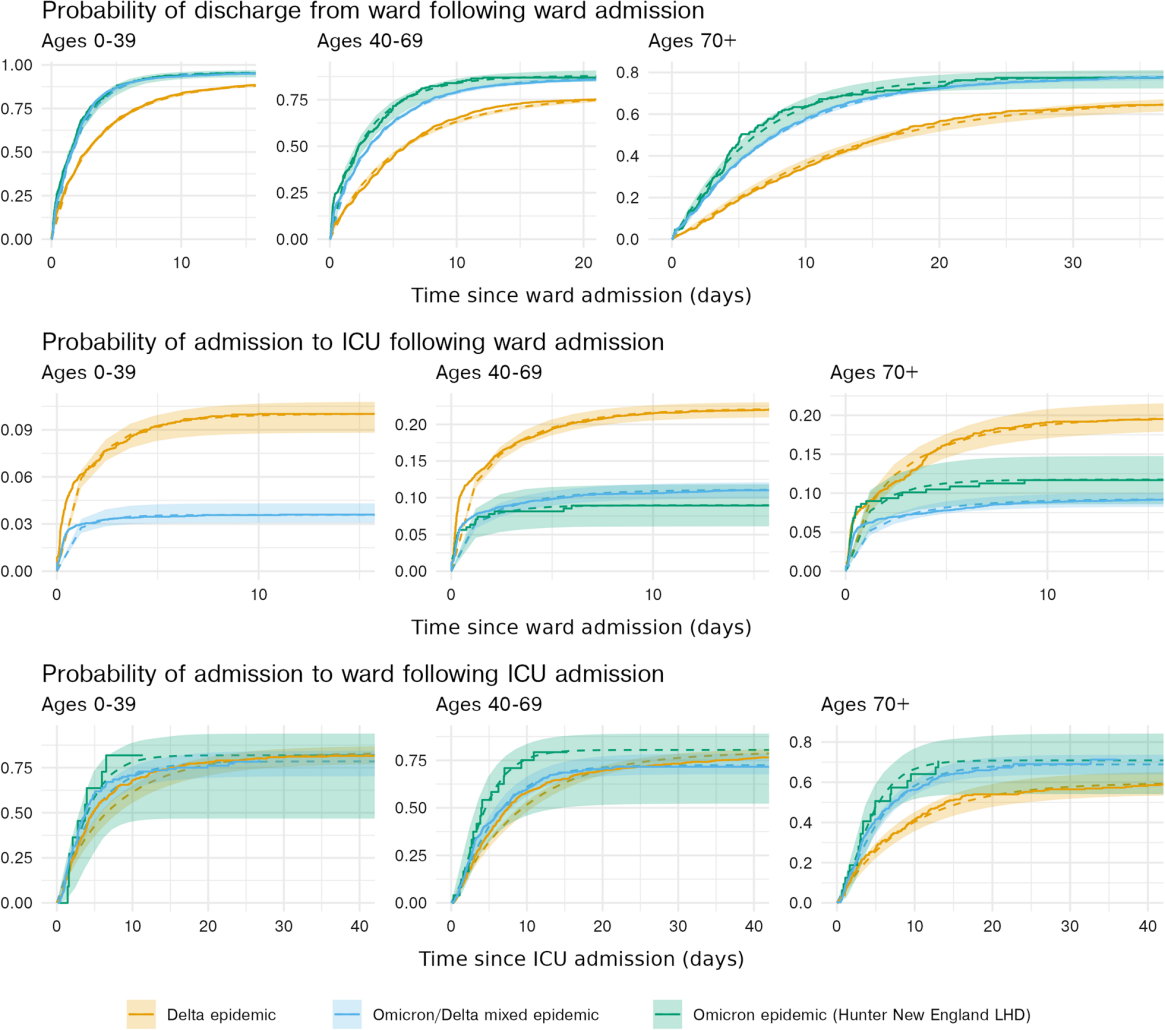
Cumulative survival probabilities of individuals in the ward-to-discharge, ward-to-ICU and ICU-to-post-ICU pathways, across epidemic periods and age groups. Solid lines represent observed data via Aalen-Johansen non-parametric estimates. Dashed lines and shaded regions represent the fit mixture distribution model means and 95% confidence intervals respectively. Note that y- and x-axis extents differ across both pathways and age groups. Delta estimates are produced over individuals admitted to hospital between 1 July 2021 and 14 December 2021, Omicron and mixed Omicron-Delta estimates are produced over individuals admitted to hospital between 15 December 2021 and 7 February 2022. Estimates for the ICU-to-post-ICU pathway could not be produced from the Hunter New England Omicron epidemic in the ward-to-ICU pathway for age group 0-39 due to limited sample size

Figure 5 shows the estimated length of stay distributions compared to the non-parametric Aalen-Johansen estimates. These figures indicate that the model provides a reasonable fit to the data. The change in scale (y-axis) indicates the probability of discharge directly from ward for all age groups is higher for the Omicron-Delta and Omicron periods, compared to the Delta period. Conversely, the probability of admission to ICU from ward is substantially lower for all age groups in the Omicron-Delta and Omicron periods, compared to the Delta period. The probabilities of admission to ward following ICU are similar across each age group, though note that these estimates are based on small numbers for the Omicron period.

The sensitivity analysis shown in Additional file 1: Fig. S4 indicates that the estimated mean lengths of stay are robust to the data filtering steps implemented. The only differences of note were in the 70+ age group for the ward-to-discharge pathway during the Delta period. Filtering individuals with symptoms after admission results in a substantial reduction in mean length of stay for this age group, from approximately 20 to 12.5 days. This is suggestive of a number of individuals in this age group having long hospital stays with infection incidental to their stay, which our multi-state model does not consider.

**Fig. 6:**
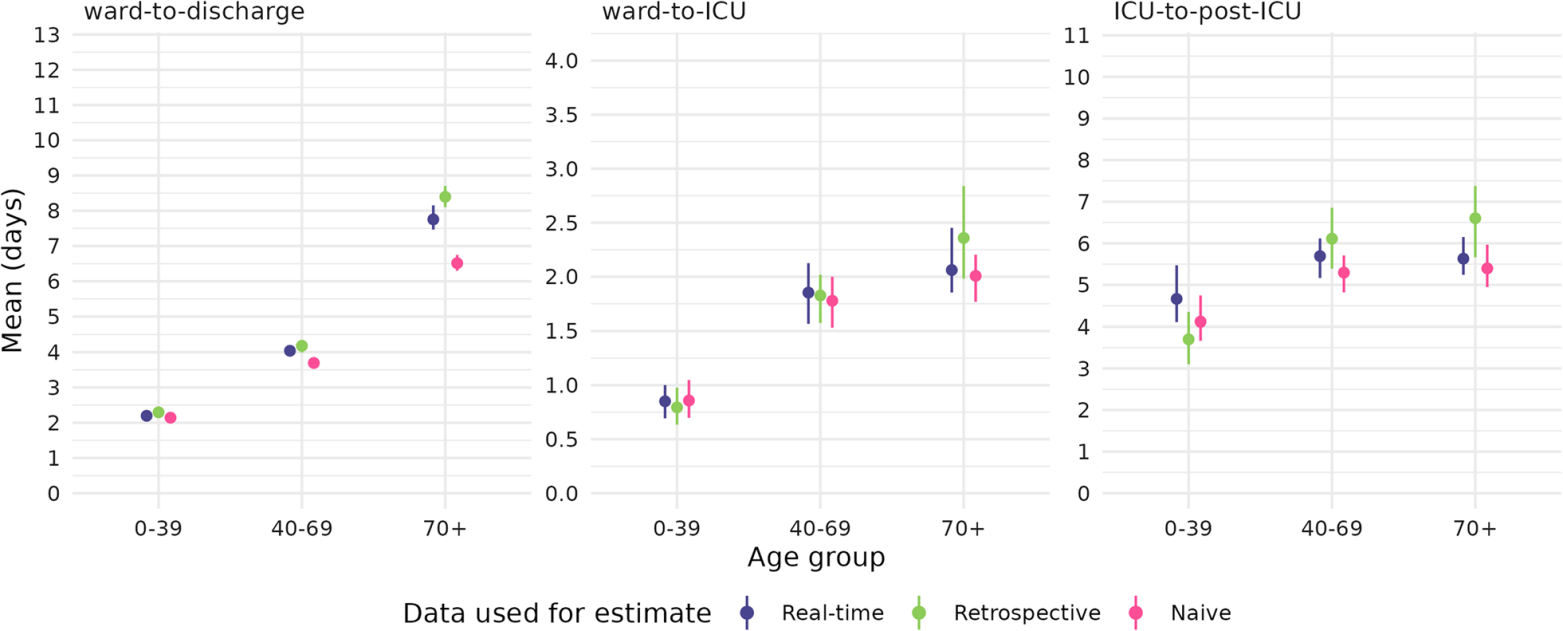
Comparison of mean length of stay (and 95% CI) for key pathways using differing estimation methods and input datasets. Real-time estimates are produced using the multi-state survival analysis framework, using data as at 7 February 2022 (as used for the main results). Retrospective estimates are produced using basic distribution fitting over fully observed patient progression, with data extracted at a later date as at 29 August 2022. Naive estimates use basic distribution fitting over real-time data as at 7 February 2022 and do not account for censoring present in the data. Mixed Omicron-Delta estimates are produced over individuals admitted to hospital between 15 December 2021 and 2 February 2022 (Note that this period differs from main results due to a change in inclusion criteria in the more recent data extract which applied to patients admitted after 3 February 2022)

Figure 6 shows the retrospective and naive estimates of the length of stay during the mixed Omicron-Delta period, compared to the real-time estimates. The real-time estimated mean lengths of stay for the Omicron-Delta epidemic period were estimated accurately (i.e., compared to the retrospective estimates), with only slight differences. The most substantial difference can be noted in the ward-to-discharge pathways, where the estimated mean length of stay was approximately 5–10% greater in the retrospective estimates, particularly in the 70+ age group. These underestimates of the mean length of stay suggest that our real-time analyses did not completely capture the tails of the length of stay distributions. The naive estimates of the length of stay are systematically shorter than the real-times estimates, particularly evident in the ward length of stay for 70+ year olds, with greater variability in the ward-to-ICU and ICU length of stay estimates consistent with the smaller numbers of observations. This systematic difference is expected, as those with longer lengths of stay are more likely to have been excluded from this analysis.

**Table 1:** Number of patients observed (n), modelled mean and 90th percentile length of stay (95% confidence intervals) across the ward-to-discharge, ward-to-ICU and ICU-to-post-ICU pathways for all age groups

Pathway	Period	Age group	n	Mean	90% Quantile
Ward to discharge					
		0–39	2521	3.60 (3.48, 3.81)	8.82 (8.47, 9.18)
	Delta	40–69	2555	5.78 (5.59, 5.99)	13.76 (13.08, 14.29)
		70+	811	12.31 (11.75, 12.95)	27.08 (25.41, 28.56)
		0–39	4208	2.16 (2.12, 2.21)	5.03 (4.83, 5.21)
	Omicron-Delta	40–69	3,014	3.93 (3.78, 4.07)	9.59 (9.10, 9.95)
		70+	2701	7.61 (7.31, 8.01)	17.36 (16.43, 18.37)
		0–39	293	2.05 (1.80, 2.30)	4.89 (4.48, 5.60)
	Omicron (HNE)	40–69	238	2.92 (2.50, 3.67)	7.21 (6.25, 8.54)
		70+	197	6.02 (4.91, 7.01)	13.46 (11.53, 16.61)
Ward to ICU					
		0–39	282	1.64 (1.41, 1.90)	4.31 (3.62, 5.13)
	Delta	40–69	733	2.07 (1.89, 2.24)	5.48 (5.10, 5.94)
		70+	239	2.90 (2.45, 3.23)	7.54 (6.45, 8.76)
		0–39	162	0.82 (0.67, 0.97)	2.17 (1.68, 2.55)
	Omicron-Delta	40-69	402	1.81 (1.54, 2.08)	4.95 (4.27, 5.56)
		70+	356	2.03 (1.83, 2.42)	5.51 (4.60, 6.30)
		0–39	12	–	–
	Omicron (HNE)	40–69	25	0.98 (0.47, 1.46)	2.66 (1.50, 5.27)
		70+	32	1.27 (0.82, 1.97)	3.32 (1.79, 6.00)
ICU to post-ICU					
		0–39	234	7.50 (6.99, 8.33)	18.07 (16.80, 19.64)
	Delta	40–69	581	9.44 (8.81, 10.07)	21.50 (20.49, 23.07)
		70+	143	8.94 (7.80, 9.91)	20.62 (18.67, 22.64)
		0–39	120	4.65 (4.11, 5.45)	10.37 (9.29, 11.46)
	Omicron-Delta	40–69	271	5.67 (5.15, 6.10)	12.04 (11.23, 13.21)
		70+	221	5.63 (5.25, 6.15)	11.97 (11.07, 13.25)
		0–39	9	3.93 (2.58, 5.68)	7.92 (5.46, 10.57)
	Omicron (HNE)	40–69	19	4.30 (3.29, 5.72)	8.50 (6.90, 11.04)
		70+	22	4.36 (3.40, 5.57)	8.58 (6.81, 10.44)

Delta estimates are produced over individuals admitted to hospital between 1 July 2021 and 14 December 2021, Omicron and mixed-Omicron-Delta estimates are produced over individuals admitted to hospital between 15 December 2021 and 7 February 2022. For each of the Delta, Omicron-Delta, and Omicron (HNE) analyses, there were (3, 1128, 6) censored individuals in the ward, (0, 4, 114) in the ICU, and (1, 135, 10) in the post-ICU ward, respectively

## Discussion

The reduced length of stay estimates we have produced are consistent with reports of Omicron-dominated epidemics in other settings internationally, for example: An analysis of a private hospital network in South Africa across 2351 Omicron patients and 6,342 Delta patients showed a reduction in median total length of stay from approximately 7–8 days in the Delta epidemic wave to 3 days in the Omicron epidemic [19]; an analysis of patients within a Houston healthcare network showed a reduction in median total length of stay from 5.4 days for Delta patients to 3.0 days for Omicron patients [20], and; an analysis of a Portuguese cohort reported a 4 day reduction in the mean length of hospital stay between Delta and Omicron [21].

We found that, across all of the Delta, mixed Delta-Omicron and Omicron Hunter New England analyses, the length of stay in ward increased with age group, a pattern which has been reported elsewhere [17, 22]. However, this trend is not observed to the same extent for the ICU pathways, with only moderate increases in the 40–69 and 70 + age-groups compared to those aged 0–39. There is no clear mechanistic explanation for this difference in trend between ward and ICU, though it may be a form of selection bias where those who are admitted to the ICU are triaged conditional on them having a greater chance of benefiting (e.g., more likely to survive, may recover at a greater rate), especially where ICU resources are relatively scarce [23]. However, there have been substantial differences in the vaccination rollout by age group in Australia that may confound this, with a general prioritisation of those at most risk of severe disease for both the first two doses and the third dose.

Although there is now strong evidence that Omicron leads to less severe disease at both the individual and population level relative to Delta [19–21, 24, 25], other factors that influence length of stay must be considered when interpreting the results presented here. At the time of analysis, ward bed occupancy in New South Wales for the mixed-Delta-Omicron epidemic had reached more than double the peak occupancy reported during the Delta-only epidemic (Fig. 1). While data was not available on the number of unoccupied beds at specific hospitals in New South Wales, prior research indicates that increased pressure on health systems can lead to self-regulation where earlier discharges will be more likely [26], which would be reflected in our results as a shorter length of stay.

It has been suggested that vaccination may result in a reduced length of stay [27], though evidence in this regard is limited. As such, the reduced length of stay observed between the two epidemic periods could be—at least partially—a result of the changes in vaccination coverage. At the start of the Delta epidemic period (1 July 2021), 6.5% of the total New South Wales population had received two doses of a COVID-19 vaccine, increasing to 52.4% mid-period (1 October 2021), and reaching a coverage of 75.0% by 14 December 2021. The booster/third dose program commenced around late-November 2021, with 24.5% of the total population having received a third/booster dose by 7 February 2022. Population immunity from prior infection at the time of Omicron emergence in New South Wales was negligible, with the count of cumulative confirmed cases reaching only approximately 1% of the population size (with very high levels of case ascertainment achieved through intensive contact tracing).

Within the epidemic periods where Omicron was present, the BA.1 PANGO lineage of SARS-CoV-2 was responsible for the majority of case growth. The BA.2 lineage of Omicron was first detected in early January 2022 [3, 28], reaching substantial prevalence in late February (making up 23% of sequenced cases across Australia across the period 14 February through to 28 February 2022 [7]). It is therefore possible that the length of stay estimates for the mixed Omicron-Delta and Omicron Hunter New England epidemic periods are influenced by the inclusion of a small number of BA.2 lineage cases.

Across the study period (1 July 2021–7 February 2022), the Australian Government approved a number of new treatments for COVID-19, including 3 monoclonal antibody treatments, 2 antivirals and 1 immunosuppressive [29]. The treatment casirivimab-imdevimab (Ronapreve, approved 15 Oct 2021) has been associated with a lower length of stay in hospital (though not in ICU) [30] and tocilizumab (Actemra, approved 1 Dec 2021) has been associated with both shorter hospital and ICU stay [31]. However, casirivimab-imdevimab has been noted to have substantially lower efficacy against the Omicron variant [32], and the use of tocilizumab was likely limited at the time due to supply constraints [33]. We were unable to incorporate the potential effects of treatment in our estimates of length of stay as no data was available in this regard. If data on treatments at the individual-level were available, this could be included as an additional stratification alongside age-group and epidemic period, allowing for the effect of treatments at the population-level to be monitored in real-time. Furthermore, when coupled with information on future availability of treatments, such outputs would potentially allow for forecasts to be produced to assist in policy decision-making regarding the optimal allocation of treatments in reducing the healthcare burden of hospitalised cases.

Our retrospective and naive analyses (Fig.  6) highlight the benefits of applying robust statistical methods that allow inclusion of all available data, as is particularly important during developing infectious disease outbreaks. Real-time estimation of length of stay is challenging and the uncertainty resulting from the censoring of outcomes means that any real-time estimates will be imperfect. The retrospective analysis shown in Figure 6 demonstrates that the multi-state model provides substantially more robust inference in real-time than the naive approach, and generates estimates that largely overcome the challenges posed by real-time estimation. The length of stay means we modelled in real-time were at most 5–10% different from those finally observed, similar to that reported in a previous study of COVID-19 hospital length of stay, which also used a multi-state survival model [11]. The hospital length of stay is key to characterising the healthcare burden (i.e., how many beds required per day) during an epidemic. In the context of providing evidence for decision makers, systematic underestimates of the length of stay such as those produced by the naive analysis will lead to underestimation of the necessary resources required to manage the healthcare burden.

## Conclusions

The results presented from hospitalised COVID-19 patients in New South Wales, Australia, indicate a reduced mean length of stay of the Omicron variant of SARS-CoV-2 compared to the Delta variant. The observed survival curves and 90% quantile estimates of length of stays are similarly reduced, showing that the overall distributions of length of stay are reduced, and not just the central tendencies. Our analysis accounted for the censoring of patient progression that is inherent to real-time data through utilisation of a competing-risk survival analysis framework. Estimates produced retrospectively using complete data aligned closely with those produced from censored data, demonstrating that this framework is a robust approach to estimating hospital length of stay in real-time.

Our use of real-time data allowed for up-to-date estimates of hospital length of stay to be rapidly reported for planning and modelling purposes. This method was utilised to provide estimates of length of stay to public health authorities throughout the Omicron epidemic in Australia, informing early response planning during a time of substantial uncertainty.

## Supplementary Information

**Additional file 1: Table S1**. Mixed Omicron-Delta epidemic period length of stay and transition probability parameter estimate means and 95% confidence intervals, with sample size (n) and correlation (Cor.) between the natural logarithms of the estimated shape and scale parameters.** Figure S1**: Mixed Omicron-Delta epidemic period length of stay parameter samples, demonstrating the correlation between log(shape) and log(scale).** Figure S2**: Cumulative survival probabilities of individuals by each pathway, across different epidemic periods and age groups. Solid lines represent observed data via Aalen-Johansen non-parametric estimates. Dashed lines and shaded regions represent the fit mixture distribution model means and 95% confidence intervals respectively. Delta estimates are produced over individuals admitted to hospital between 1 July 2021 and 14 December 2021, Omicron and mixed-Omicron-Delta estimates are produced over individuals admitted to hospital between 15 December 2021 and 7 February 2022. Estimates for the ICU-to-post-ICU pathway could not be produced from the Hunter New England Omicron epidemic in the ICU and post-ICU pathways due to limited sample counts in the data.** Figure S3**: Cumulative survival probabilities of individuals by each pathway, across different epidemic periods and age groups. Solid lines represent observed data via Aalen-Johansen non-parametric estimates. Dashed lines and shaded regions represent the fit mixture distribution model means and 95% confidence intervals respectively. Delta estimates are produced over individuals admitted to hospital between 1 July 2021 and 14 December 2021, Omicron and mixed-Omicron-Delta estimates are produced over individuals admitted to hospital between 15 December 2021 and 7 February 2022. Estimates for the ICU-to-post-ICU pathway could not be produced from the Hunter New England Omicron epidemic in the ICU and post-ICU pathways due to limited sample counts in the data.** Figure S4**: Sensitivity analysis across differing degrees of filtering during construction of the clinical datasets. Estimated length of stay means and 95% confidence intervals shown. For the ‘No filtering’ and ‘Filtering out symptom onset after admission’ scenarios, individuals with episodes greater than 5 days apart were still removed. Data as of 2022-01-25.

## Data Availability

The secondary dataset that supports the findings of this study is available from New South Wales Ministry of Health but restrictions apply to the availability of this dataset, which was used under license for the current study, and as such the dataset has not been made publicly available. This dataset is not available upon request from the authors, but may be available upon request from the New South Wales Ministry of Health. All code required to produce the results is available at 10.17605/OSF.IO/EA8Z5, with the original source control repository available at github.com/ruarai/los_analysis_competing_risks/. Full tables of estimated parameters for all pathways, age-groups and epidemic periods are available for download at 10.17605/OSF.IO/UNS7V.
