# Supplementary material for: Real-time analysis of hospital length of stay in a mixed SARS-CoV-2 Omicron and Delta epidemic in New South Wales, Australia
Source: BMC Infect Dis. 2023 Jan 17;23:28. doi: 10.1186/s12879-022-07971-6 (PMC9844941; doi:10.1186/s12879-022-07971-6)
Supplement: Supplementary file 1 — Additional file 1: Table S1. Mixed Omicron-Delta epidemic period length of stay and transition probability parameter estimate means and 95% confidence intervals, with sample size (n) and correlation (Cor.) between the natural logarithms of the estimated shape and scale parameters. Figure S1: Mixed Omicron-Delta epidemic period length of stay parameter samples, demonstrating the correlation between log(shape) and log(scale). Figure S2: Cumulative survival probabilities of individuals by each pathway, across different epidemic periods and age groups. Solid lines represent observed data via Aalen-Johansen non-parametric estimates. Dashed lines and shaded regions represent the fit mixture distribution model means and 95% confidence intervals respectively. Delta estimates are produced over individuals admitted to hospital between 1 July 2021 and 14 December 2021, Omicron and mixed-Omicron-Delta estimates are produced over individuals admitted to hospital between 15 December 2021 and 7 February 2022. Estimates for the ICU-to-post-ICU pathway could not be produced from the Hunter New England Omicron epidemic in the ICU and post-ICU pathways due to limited sample counts in the data. Figure S3: Cumulative survival probabilities of individuals by each pathway, across different epidemic periods and age groups. Solid lines represent observed data via Aalen-Johansen non-parametric estimates. Dashed lines and shaded regions represent the fit mixture distribution model means and 95% confidence intervals respectively. Delta estimates are produced over individuals admitted to hospital between 1 July 2021 and 14 December 2021, Omicron and mixed-Omicron-Delta estimates are produced over individuals admitted to hospital between 15 December 2021 and 7 February 2022. Estimates for the ICU-to-post-ICU pathway could not be produced from the Hunter New England Omicron epidemic in the ICU and post-ICU pathways due to limited sample counts in the data. Figure S4: Sensitivity analysis across d [file 12879_2022_7971_MOESM1_ESM.pdf]

Supplementary materials

Pathway	Age	n	Scale		Shape		Median		Cor.	Probability	
ward-to-discharge	0-39	4208	2.27	[2.16, 2.39]	0.95	[0.92, 0.98]	1.46	[1.42, 1.51]	-0.85	0.96	[0.95, 0.96]
	40-69	3014	4.98	[4.68, 5.30]	0.79	[0.76, 0.83]	2.45	[2.35, 2.54]	-0.83	0.87	[0.86, 0.88]
	70+	2701	7.32	[6.71, 7.95]	1.04	[0.99, 1.09]	5.36	[5.16, 5.58]	-0.88	0.78	[0.76, 0.80]
ward-to-ICU	0-39	162	1.48	[1.09, 1.93]	0.56	[0.46, 0.67]	0.41	[0.31, 0.52]	-0.67	0.04	[0.03, 0.04]
	40-69	402	3.81	[3.12, 4.61]	0.48	[0.43, 0.53]	0.80	[0.66, 0.94]	-0.61	0.11	[0.10, 0.12]
	70+	356	4.09	[3.34, 4.95]	0.50	[0.45, 0.57]	0.92	[0.77, 1.10]	-0.67	0.09	[0.08, 0.10]
ward-to-death	all	289	41.69	[32.20, 53.31]	0.82	[0.72, 0.93]	21.35	[18.35, 24.93]	-0.80	0.03	[0.03, 0.03]
ICU-to-discharge	0-69	62	10.71	[5.86, 17.52]	0.70	[0.52, 0.92]	4.15	[2.62, 6.13]	-0.66	0.13	[0.10, 0.16]
	70+	25	10.71	[5.86, 17.52]	0.70	[0.46, 1.02]	4.15	[2.16, 7.35]	-0.51	0.08	[0.06, 0.11]
ICU-to-death	0-69	48	20.60	[13.91, 29.24]	0.96	[0.74, 1.23]	13.20	[9.20, 18.00]	-0.63	0.13	[0.10, 0.17]
	70+	55	20.60	[13.91, 29.24]	0.86	[0.67, 1.10]	11.27	[8.22, 14.62]	-0.69	0.23	[0.19, 0.28]
ICU-to-post-ICU	0-69	391	4.15	[3.58, 4.73]	1.30	[1.16, 1.46]	4.08	[3.69, 4.49]	-0.77	0.74	[0.70, 0.78]
	70+	221	4.15	[3.58, 4.73]	1.37	[1.21, 1.54]	4.37	[3.87, 4.95]	-0.65	0.69	[0.63, 0.74]
post-ICU-to-discharge	0-39	110	4.34	[3.58, 5.20]	1.12	[0.94, 1.34]	3.50	[2.88, 4.19]	-0.64	0.97	[0.90, 0.99]
	40-69	209	4.34	[3.58, 5.20]	1.63	[1.41, 1.88]	5.66	[4.98, 6.45]	-0.76	0.93	[0.85, 0.97]
	70+	134	4.34	[3.58, 5.20]	2.00	[1.70, 2.34]	7.26	[6.38, 8.30]	-0.75	0.77	[0.69, 0.84]
post-ICU-to-death	all	24	15.97	[1.44, 62.61]	0.95	[0.42, 1.84]	6.60	[1.87, 15.74]	-0.88	0.05	[0.05, 0.05]

Table S1: Mixed Omicron-Delta epidemic period length of stay and transition probability parameter estimate means and 95% confidence intervals, with sample size (n) and correlation (Cor.) between the natural logarithms of the estimated shape and scale parameters. Note that ward-to-death and post-ICU-to-death probability estimates are produced using the Aalen-Johansen non-parametric estimator and are not displayed. * Note that the probabilities reported here are estimated as described in the Methods section in the main text, and are not adjusted for co-morbidities, vaccination status, prior infection with SARS-CoV-2, sex, or other variables associated with severity of disease. They do not represent a formal analysis of Omicron severity — they represent estimates of the observed transition probabilities during the mixed Omicron-Delta epidemic in the largely previously uninfected study population. We caution against generalising these values to other settings, particularly with different demographics, vaccination coverage, or prevalence of comorbidities, or assuming they would remain constant in other time-periods in the study population.

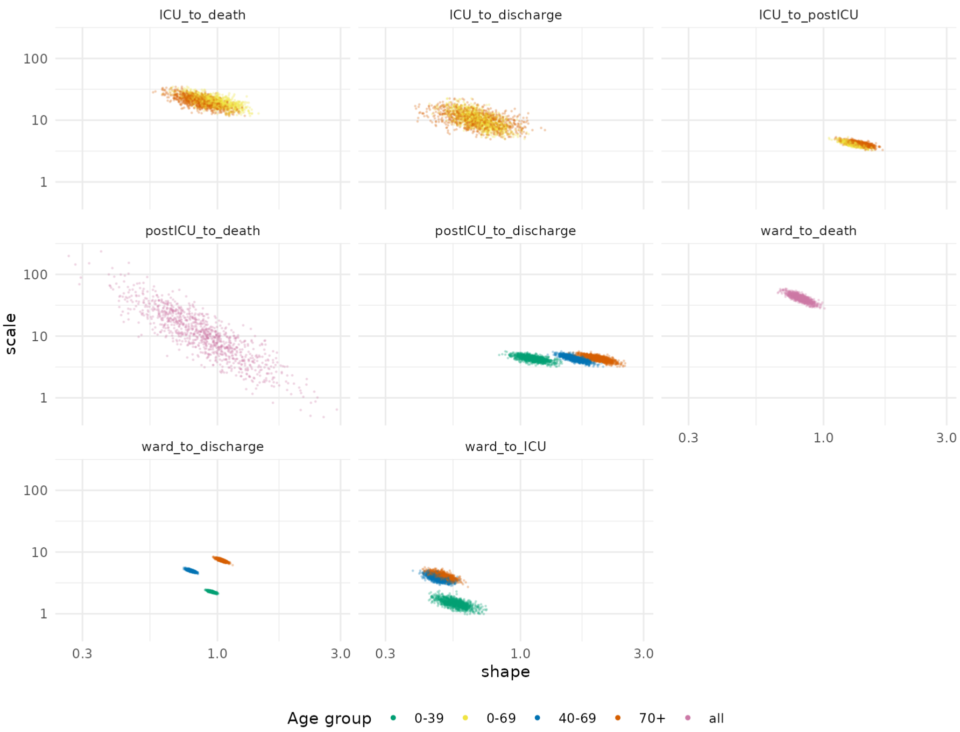

Figure S1: Mixed Omicron-Delta epidemic period length of stay parameter samples, demonstrating the correlation between log(shape) and log(scale).

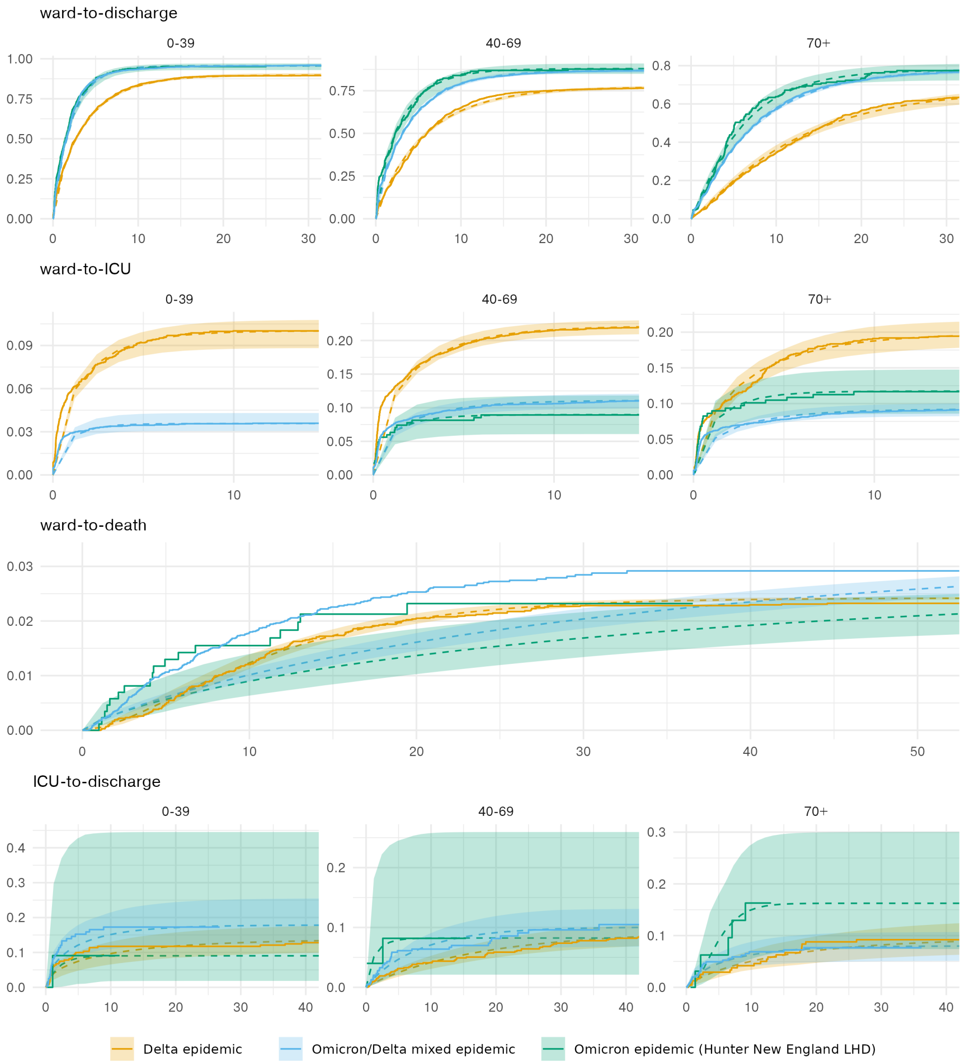

Figure S2: Cumulative survival probabilities of individuals by each pathway, across different epidemic periods and age groups. Solid lines represent observed data via Aalen-Johansen non-parametric estimates. Dashed lines and shaded regions represent the fit mixture distribution model means and 95% confidence intervals respectively. Delta estimates are produced over individuals admitted to hospital between 1 July 2021 and 14 December 2021, Omicron and mixed-Omicron-Delta estimates are produced over individuals admitted to hospital between 15 December 2021 and 7 February 2022. Estimates for the ICU-to-post-ICU pathway could not be produced from the Hunter New England Omicron epidemic in the ICU and post-ICU pathways due to limited sample counts in the data.

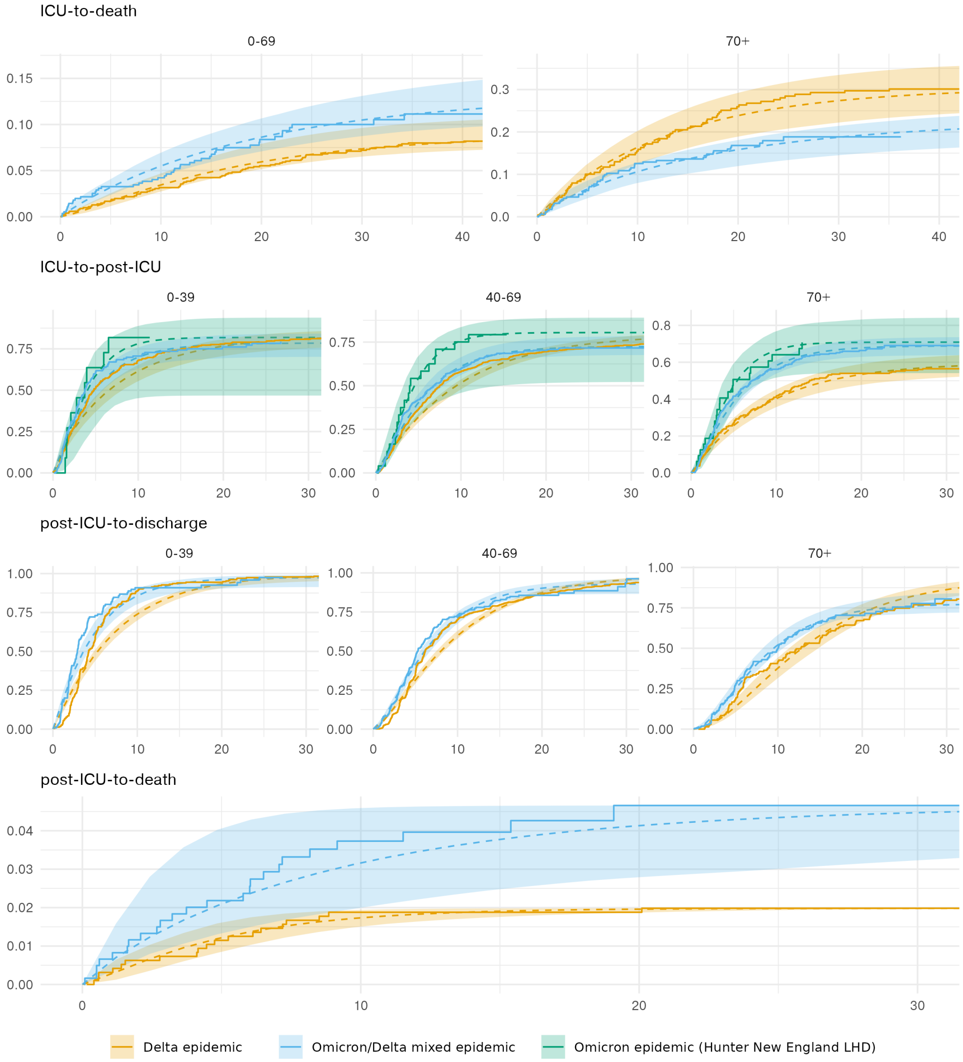

Figure S3: Cumulative survival probabilities of individuals by each pathway, across different epidemic periods and age groups. Solid lines represent observed data via Aalen-Johansen non-parametric estimates. Dashed lines and shaded regions represent the fit mixture distribution model means and 95% confidence intervals respectively. Delta estimates are produced over individuals admitted to hospital between 1 July 2021 and 14 December 2021, Omicron and mixed-Omicron-Delta estimates are produced over individuals admitted to hospital between 15 December 2021 and 7 February 2022. Estimates for the ICU-to-post-ICU pathway could not be produced from the Hunter New England Omicron epidemic in the ICU and post-ICU pathways due to limited sample counts in the data.

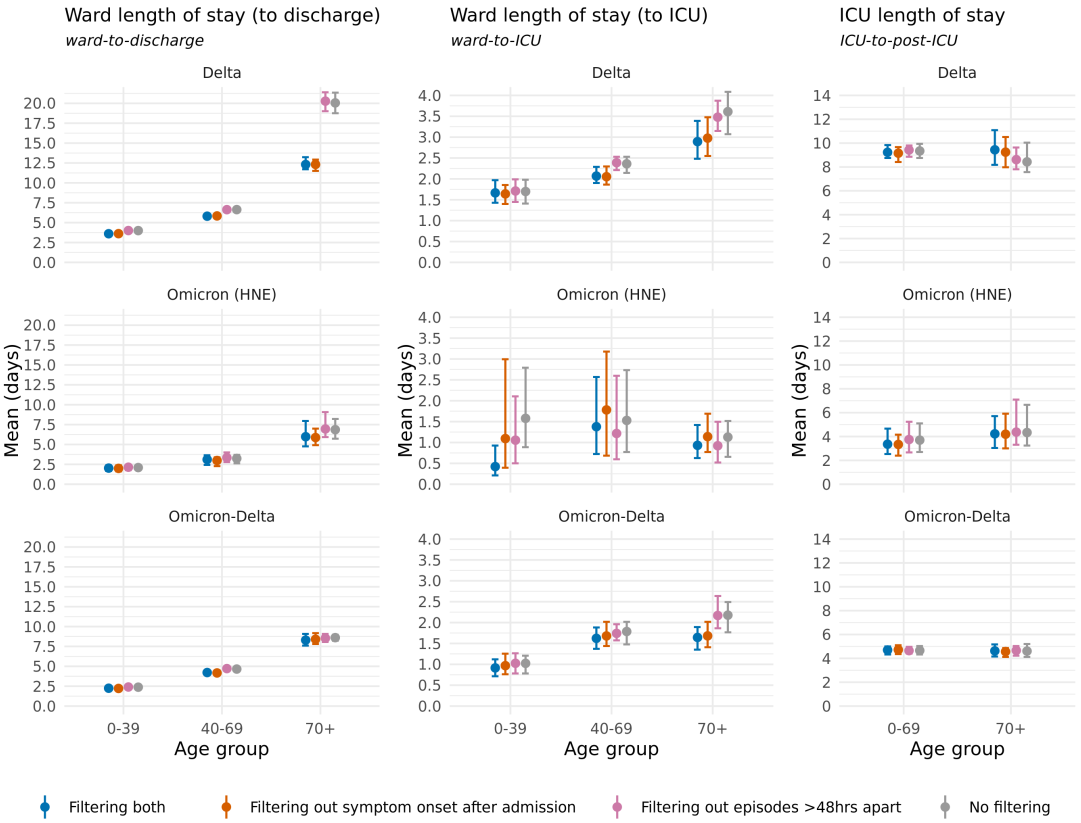

Figure S4: Sensitivity analysis across differing degrees of filtering during construction of the clinical datasets. Estimated length of stay means and 95% confidence intervals shown. For the 'No filtering' and 'Filtering out symptom onset after admission' scenarios, individuals with episodes greater than 5 days apart were still removed. Data as of 2022-01-25.
